# Autism-Friendly Healthcare: A Narrative Review of the Literature

**DOI:** 10.7759/cureus.64108

**Published:** 2024-07-08

**Authors:** Samar Z Hamdan, Amanda Bennett

**Affiliations:** 1 Department of Pediatrics, College of Medicine and Surgery, King Abdulaziz University, Jeddah, SAU; 2 Division of Developmental and Behavioral Pediatrics, Children's Hospital of Philadelphia, Perelman School of Medicine at the University of Pennsylvania, Philadelphia, USA

**Keywords:** autistic patients, healthcare experience, barriers, autism-friendly, healthcare, autism

## Abstract

Disparities in healthcare access, delivery, and outcomes exist between autistic and non-autistic individuals. Autism-friendly healthcare initiatives aim to facilitate and improve the healthcare experience of autistic individuals by addressing commonly encountered challenges. While there is no consensus regarding the definition of autism-friendly healthcare, in this narrative review, we examine previously published research to determine the most important components of autism-friendly healthcare. Patient-related factors, provider-related factors, and system-related factors should be addressed. Proactivity, flexibility, and collaboration should guide the process of transforming the healthcare system. Finally, multiple strategies can be utilized as appropriate to the setting and individuals.

## Introduction and background

Autism spectrum disorder (ASD) is a prevalent neurodevelopmental disorder worldwide. The core features of ASD include challenges with communication and social interactions, a pattern of repetitive behaviors, inflexibility and insistence on sameness, narrow and fixated interests, and/or hypo- or hypersensitivities [1]. However, autism's effect on an individual's health extends far beyond its core features with high rates of behavioral, psychiatric, and medical co-occurring conditions among autistic individuals [2-5]. Accordingly, autistic individuals have high rates of healthcare service utilization across their life span and across various service areas, although variable based on age, abilities, and comorbidities [6-9].

Unfortunately, higher utilization of healthcare services has not been translated into high-quality care or optimal health. On the contrary, current literature suggests suboptimal care practices, lower satisfaction with services and providers [10-12], and poor health quality among autistic patients [13]. For instance, evidence suggests challenges with providing important aspects of preventative care to autistic individuals [14]. Among autistic adults, lower rates of tetanus vaccination and Papanicolaou smears for cervical screening have been documented [8,11]. Based on an online survey developed by the Autism Spectrum Disorders in the European Union, less than 40% of autistic adults and their caregivers reported routine visits for vision assessment, hearing screening, breast exams (for women), or sexual health maintenance [15]. Similarly, it has been shown that autistic youth are less likely to receive preventive services such as vaccinations despite high contact rates with their medical providers [6]. Also, autistic youth are less likely to complete depression and suicide screening compared to non-autistic adolescents [16]. Additionally, healthcare utilization by autistic individuals differs from that of the general population in other ways. Autistic youth are 30 times more likely to present to the emergency department (ED) compared to non-autistic youth, with a higher likelihood of repeat visits and admissions [17]. Also, among autistic children admitted for inpatient care, the proportion of hospitalization for ambulatory care-sensitive conditions, which are potentially preventable, is higher than non-autistic children [18]. Disparities in outcomes have also been documented. Autistic individuals frequently report delayed presentation for care and untreated health issues [19]. Moreover, clinical and population-based studies indicate higher rates of mortality among autistic individuals [20-22].

This mismatch between higher utilization of healthcare services and worse healthcare outcome among autistic individuals is alarming. While biological susceptibility might be a contributing factor to the unfavorable outcome, failure of the healthcare system to meet the needs of this vulnerable population is an essential one [23]. Poor access to care and suboptimal delivery have been documented [19,24-26]. Accordingly, accommodations and modifications are considered helpful in building a positive healthcare experience for autistic patients [27]. Likewise, clinicians question the ability of the current system to support autistic individuals [28]. Therefore, improving the healthcare system to meet the needs of this population is fundamental. Certainly, the autistic community has identified the development of autism-friendly healthcare as a research priority [29].

While evidence-based standards to guide the care for autistic individuals in the healthcare setting are lacking, extensive work has been done to examine and understand their current experience as a first step toward improvement. Based on the reported barriers and facilitators of care among autistic patients, recommendations to enhance healthcare access and delivery have been made and applied in multiple settings with the goal of making the healthcare environment and processes more "autism-friendly." Although there is no consensus on the definition of "autism-friendly healthcare" in the literature, many quality improvement initiatives are using the terminology to describe the process of identifying difficulties faced by the autistic population when engaging with the healthcare system and making adaptations to remove these barriers and facilitate participation, with ongoing work to systemically identify its components and understand how to apply them in multiple healthcare settings [30]. In this narrative review of the literature, we aim to present the current status of the healthcare system as experienced by the autistic population and a guide to transform it into an autism-friendly system from the perspectives of autistic patients, their caregivers, and healthcare providers across disciplines and settings. Based on available literature, we will answer three important questions required to lead this transformation: why, what, and how. While there might be some differences between pediatric and adult care among autistic patients, there are common themes (e.g., insufficient physician training and cost of service) [31]. Thus, we chose to include relevant data for pediatric and adult care in this review. Of note, we will adopt an identity-first language when referring to autistic patients in this review to match the preference of the majority of the autistic community [32].

## Review

Toward autism-friendly healthcare

While there are common barriers to healthcare access and delivery experienced by individuals with different types of disabilities, autistic individuals face additional distinctive patterns of autism-related challenges [26]. Barriers have been recognized at different stages of patients' interaction with the healthcare system such as access to care (e.g., identifying a primary care provider or scheduling an appointment) [33], waiting for care [28,34], establishing rapport with a provider [19], going through a diagnostic evaluation (e.g., examination) [34], and following instructions after care (e.g., following through for a referral) [34]. Although general measures adopted to increase healthcare accessibility might support the autistic population, targeted and specific adjustment is required [26]. When surveyed, more than half of autistic participants who received medical care without accommodations stated that adjustments would have been necessary and/or helpful [35]. Additionally, 45% of participants attributed missing appointments to poor access and lack of adjustments [35]. Fortunately, healthcare providers also recognize the necessity for and the feasibility of healthcare adjustment [28]. To guide the transformation of the healthcare system into an autism-friendly system, we will present here major barriers to healthcare access and delivery, important facilitators to address current challenges, goals to aim for achieving through change, principles to guide the change, and intervention strategies that could be applied and implemented, as summarized in Table 1. Ultimately, the goal should be to improve access, improve healthcare delivery across the care continuum, and improve health outcomes.

**Table 1:** Components of autism-friendly healthcare

Autism-friendly healthcare		
Why	Increased access to healthcare, provision of high-quality evidence-based care, improved healthcare experiences, and improved health-related outcomes	
What	Factors to address	Goals to achieve
	Patient-level factors: Challenges with communication, sensory differences, and insistence on sameness and intolerance of change	Patient-focused goals: Improved communication, optimization of the sensory experience and environment, increased predictability, and maintained continuity and consistency
	Provider-level factors: Lack of knowledge, low self-efficacy, and stereotyped beliefs about autism	Provider-focused goals: Autism-specific education and training for healthcare providers
	System-level factors: Logistical and operational factors, financial and economic factors, and social and societal factors	System-focused goals: Simplified healthcare systems (access and care delivery), improved insurance policies, formal support programs, and increased autism awareness and acceptance among the general population
How	Guiding principles	Intervention strategies
	Proactivity and advanced planning, flexibility and individualization, and collaboration	Data collection and communication tools (Health Passport), packages of interventions (clinical pathway), specialized health check or primary care visits, medical home or specialized primary care facilities, environmental and architectural design, behavioral strategies, and technology-based strategies

Factors affecting care access and delivery (barriers and facilitators)

Patient-level factors, provider-level factors, and system-level factors have been identified as contributing to the challenges faced by autistic people in the healthcare system [36]. However, while it might be useful to adopt this categorization to facilitate the conceptualization and implementation of change, it is important to recognize that most factors are complex and result from the interaction between patients, providers, and the system. Nevertheless, the first step toward autism-friendly healthcare is to recognize, understand, and address these factors. 

Patient-Level Factors

Many autistic traits and associated comorbidities affect healthcare access and delivery among autistic patients in several ways, as summarized in Table 2. However, major patient-level factors that will be discussed here with possible solutions include challenges with communication and social interactions, sensory differences, and insistence on sameness and intolerance of change.

**Table 2:** Autism symptoms associated with challenges in the healthcare system

Core features of autism	Examples of associated challenges in the healthcare system
Deficit in social communication and social interaction	Difficulties with answering open-ended and subjective questions during medical interviews. Difficulties with describing pain and internal physical changes. Mismatch between spoken words and non-verbal means of communication and self-expression. Difficulties with requesting and scheduling appointments, especially by phone
Stereotyped or repetitive behaviors	Literal interpretation of questions and instructions. Fear of experiencing stigmatization in the healthcare setting due to repetitive movements and behaviors. Repetitive questioning
Insistence on sameness, inflexibility, or distress with change	Disturbance of usual routine due to medical appointments or inpatient hospitalizations. Challenges with high providers' turnover in academic facilities
Highly restricted, fixated interests	Fixation on certain symptoms, aspects of care, or medical instruments
Hyper- or hyperreactivity sensory input	Challenges with highly stimulating healthcare environments such as waiting rooms and acute care settings. Challenges with examinations and tests that require physical contact with providers or instruments. Challenges with medications with certain texture, smell, or taste. Variable processing and expression of pain leading to delayed or missed diagnosis
Additional features and comorbidities	Examples of associated challenges in the healthcare system
Language impairment	Challenges with verbal communication and need to use alternative means of communication
Slow processing speed	Difficulties with high-speed communication in healthcare settings. Difficulties with high-speed processes and procedures of care delivery
Challenges with executive function	Challenges with prioritization and summarization of symptoms and health issues. Challenges with planning for future appointments. Challenges with remembering appointment details or taking medications
Anxiety	Traumatizing healthcare experiences leading to avoidance. Anxiety might exacerbate communication difficulties

Challenges with communication and social interactions: Patient-provider communication challenges negatively affect healthcare access and delivery among autistic individuals as reported by autistic patients, their caregivers, and healthcare providers [19,25,26,33,36-39]. Autistic individuals commonly describe that they don't feel understood by their physicians [19]. On the other hand, only a small minority of healthcare providers report high levels of confidence in communicating with autistic patients [38].

Both expressive and receptive communication challenges can hinder patient-provider interactions during healthcare encounters [33]. For instance, autistic individuals might lack the ability to communicate using spoken language and rely on alternative and augmentative ways of communication [25]. However, autistic individuals with more advanced verbal abilities and social skills also experience issues related to communication. Literal interpretation of questions and instructions [37], mismatch between spoken words and non-verbal means of communication, difficulties with answering open-ended questions [34,39], and decreased likelihood of unprompted spontaneous volunteering of information [40] can affect communication in the healthcare setting. For example, very broad, open-ended, and subjective questions (e.g., how are you doing?) might lead to the use of scripted learned responses rather than generating informative answers [33,40]. Additionally, autistic individuals might struggle with describing their internal state and expressing pain or other symptoms which can hinder the diagnostic process, especially if providers fail to adapt their information-gathering techniques to the autistic population's needs [25,28,37]. Furthermore, the high speed of communication in the healthcare setting is challenging for autistic individuals and their abilities to process essential information and make important health-related decisions [26,37,39]. Unexpected changes (e.g., new provider), anxiety, and sensory overload can increase communication failure [19], even for autistic patients who are typically verbally fluent [40,41]. 

Unfortunately, difficult communication can lead to significant consequences. Incidents of misunderstanding as well as difficulties with establishing rapport have been reported by patients and providers [25,39]. Consequences can also extend to transmitting and recording inaccurate medical information [39]. Additionally, communication difficulties might be contributing to self-reported challenges with requesting medical assistance and discussing certain topics such as mental health [19]. One of the major difficulties faced by the autistic population in the healthcare setting is communication to request and schedule appointments, with booking using the phone being exceptionally problematic [19,34,37]. Likewise, unexpected and unplanned phone calls from providers with no time for preparation might be difficult for autistic individuals [42].

Patient- and caregiver-initiated strategies, provider-adopted strategies, and system-level strategies can facilitate communication and subsequently appropriate healthcare access and delivery for autistic patients [43]. Patients and their caregivers might instinctively use alternative ways of communication such as written language, communication devices, and/or technology to express their needs and achieve their healthcare goals. For example, patients and caregivers might use health-related pictures or pre-written notes on their phones to facilitate communication with their providers [33]. They might also ask permission to make audio recordings of important discussions and recommendations [39].

At the provider level, offering and accepting the use of non-verbal ways of communication to supplement or replace verbal conversations is essential [24]. Written information, visual aids, and technology-based tools can be utilized [39]. Written questions and information can be helpful before the visit to gather information and prepare for the encounter or after the visit to share a summary of recommendations [39,44]. Visual aids such as pictures, drawing on whiteboards, and timers are particularly useful [39,45]. Additionally, providers should adjust their communication style during verbal exchanges. Providers should intentionally use clear short sentences, concrete language, direct or prompted questioning, and detailed answers and explanations [33,39,40,46]. Use of visual-verbal prompting (V-VP) during interviews, which involves asking questions about specific details of an event written on a specific prompt wheel, improved the specificity and increased the proportion of relevant information reported by autistic individuals [47]. On the contrary, providers should avoid over-dependence on open-ended and broad questions, ambiguous language, and non-verbal cues [44,46]. They should observe body language and facial expression for signs of anxiety or discomfort but should not depend on them solely to interpret or disregard information expressed and shared directly by autistic patients [25]. Providers should be sensitive to patients' eye contact preferences and consider changing their position based on patients' liking (e.g., sitting next to the patient instead of facing them) [46,48]. Providers should also accept the literal interpretation of information shared by autistic people [19,39]. Furthermore, providers should allocate more time for communication and slow down their pace to allow processing and exchange of information [25,33]. They should plan for longer pauses, anticipate more silence, and refrain from rushing patients or repeating questions as autistic individuals might take longer to give answers [40]. With a high percentage of autistic individuals reporting that they try to avoid phone calls for health-related communication [19], providers should not rely on the phone to gather or share information, although it can be used as an adjunct way of communication when necessary [42]. Additionally, autistic patients should be granted access to communicate with their providers using text-based methods (e.g., email) [19]. Providers should be prepared to communicate with autistic patients and their caregivers or advocates if needed [33]. Written instruction should also be considered for caregivers accompanying autistic patients who might have difficulties with attention as they try to support their children during medical encounters [43]. For similar reasons, post-encounter communication to ensure understanding is also encouraged [35].

System-level strategies should include offering longer or double appointments to allow more time for communication and processing of information [42,46]. Additionally, offering variable means of consultations such as telemedicine appointments in addition to face-to-face appointments should be considered [42]. Moreover, the appointment booking and registration systems should be a target for improvement. For example, online booking systems are preferred [42,44]. If phone use is inevitable, offering a direct rather than a centralized number is preferred. Additionally, the use of a screen or text-based system to check in for an appointment instead of queuing at the reception desk might be useful [46]. Finally, strategies aiming to decrease sensory stress and anxiety related to uncertainty can enhance effective communication [25].

Sensory differences: Sensory hypo- and hypersensitivities affect the health experience of autistic individuals significantly [35,37,39]. It is frequently reported as a top barrier to healthcare access and delivery by the autistic community [26,37]. In fact, autistic individuals are more likely than individuals with other disabilities to endorse sensory sensitivity as an obstacle to care [26]. Particularly, autistic individuals have challenges tolerating environments with high levels of sensory stimulation, such as waiting rooms [19,28,37], EDs [49], and inpatient units [50]. Auditory overstimulation is especially problematic in these environments [49,50]. While loud noise might be uncomfortable for autistic and neurotypical individuals, autistic individuals are more likely to find background noise stressful and exhausting [39]. However, for autistic individuals, sensory stress extends beyond auditory overstimulation to other senses. Possible visual stressors include bright light, flickering light, screens, moving objects, and highly patterned surfaces [39,40,51]. Autistic patients might perceive the environment as exceptionally stressful if the light level is out of their control such as in shared rooms [39]. Tactile aversion and sensitivity can hinder some aspects of physical examination which require physical contact with providers or instruments [37]. Olfactory stimuli can be a trigger for emotional stress and sensory overload in some autistic patients [39]. Autistic individuals might also find the sensory input from people in close proximity to them stressful [39]. Additionally, sensory overload might hinder focus, exaggerate anxiety, worsen communication, and lead to behavioral challenges such as meltdowns or shutdowns [19,26,39,40,52]. Thus, an adjusted sensory environment and a novel approach to handling sensory differences are important aspects of autism-friendly healthcare [11,35-38].

While it is neither reasonable nor obligatory to stop all sensory stimulation, efforts should aim to improve the sensory environment, optimize the sensory input, and minimize the effect of sensory differences on care [28,37,40,45]. This can be achieved through different approaches, summarized with examples in Table 3: removal of unnecessary sensory stimuli [46,51,53], substitution of highly noxious stimuli with more tolerable stimuli [51,53], reducing the impact of sensory stimuli on patients by either using a mediation or isolation strategy [39,40,51,53] or reducing exposure/contact time with sources of sensory stress [35,37,39,40,46,51], utilizing adjustable sources of sensory stimuli that allow flexibility and individualization [51,53], modification of care to adopt sensory differences [19,34], embracing sensory interests, behaviors, and strategies to facilitate care [54], and allowing time and space for recovery from sensory overstimulation [53]. Use of a sensory toolkit with multiple items such as noise-canceling headphones, light spinners, fidgets, and weighted pads is a practical strategy to achieve multiple sensory optimization goals [19,45,55]. Additionally, autism-friendly architectural design can ensure a more appropriate sensory environment [51,53].

**Table 3:** Strategies to optimize the sensory experience of autistic patients in the healthcare system

Strategies to optimize the sensory experience of autistic patients	Examples
Removal of unnecessary sensory stimuli	Avoiding scented perfumes, avoiding scented plants, avoiding reflective surfaces, avoiding decorative lighting or objects, avoiding color contrast
Substitution of highly noxious stimuli with more tolerable stimuli	Natural lighting instead of artificial lighting, LED lighting instead of florescent lighting, pastel or natural colors instead of bright colors
Reduction of the impact of sensory stimuli on patients by using a mediator or isolation strategy	False ceiling to cover lighting source, sound insulation between rooms and sound-absorbing materials inside rooms, sound-absorbing floors, use of noise-canceling headphones, use of sunglasses, optimal position of lighting source to avoid direct contact, high-level windows to limit distraction
Reduction of exposure/contact time with the sources of sensory stress	Offering private quieter waiting rooms, allowing patients to wait outside to be called directly to their appointments, strategic scheduling of appointments to avoid busy or crowded times
Use of adjustable sources of sensory stimuli that allows flexibility and individualization	Easy-to-use lighting source with accessible control switch, fans with manual operating system to avoid sudden activation
Modification of care to accommodate sensory differences	Acknowledgement of atypical experience and expression of pain during medical evaluation, modification of examination and procedures as needed
Embracement of sensory interests, behaviors, and strategies to facilitate medical care	Use of sensory items for distraction during procedures
Time and space for recovery from sensory overstimulation	Offering quiet spaces for retreat, transitional spaces between environments with different sensory experiences

Insistence on sameness and intolerance of change: Insistence on sameness and inflexibility is a core feature of ASD that leads to a significant challenge with healthcare access and delivery. To overcome this challenge, adjustments to increase predictability are often required. This can be achieved by decreasing uncertainty, increasing consistency, avoiding changes, especially if sudden or unplanned, and allowing enough time for preparation [19,37,40].

Increased predictability should start with preparatory steps before the healthcare encounter. For instance, choosing an appointment time that fits the autistic individual's schedule with minimal disturbance to the usual routine is preferable [40,46]. This can be achieved either by strategic scheduling by autistic individuals or their caregivers or through a preferential scheduling system that is flexible and adaptable. Once booked, appointments should be started on time, and if not possible, suspected delays should be communicated clearly with an option to reschedule or an adjustment to improve the waiting experience [40,46]. Additionally, preparatory information prior to any health encounter is essential and can lead to increased attendance and decreased anxiety [46]. Preparatory material, preferably supported with visual aids, should include information related to the type of visit, expected duration of the visit, personnel to be encountered during the visit, and a detailed description of the healthcare facility and how to access it [39]. Additionally, social stories can be used to communicate the sequence of a visit or procedure ahead of time [45,55]. Experimental trials, acclimatization visits, and/or virtual tours of healthcare facilities can be very helpful, especially for planned admissions or procedural visits [40,46].

During healthcare encounters, visual supports such as social storyboards, visual schedules, modeling, and role-play can be used to facilitate examination or procedures, all of which were found to be feasible and effective [45]. Step-by-step narration is also advocated [33]. Demonstrating equipment and tools and offering opportunities for exploration before using them is helpful [46]. Additionally, autistic individuals reported a need for consistency of care [37], which should include continuity of healthcare providers during an encounter (e.g., throughout an inpatient hospitalization), continuity of providers across appointments, continuity of space of care, and consistency of appointment time for recurrent appointments [40,46]. Similarly, clinicians endorse continuity of care to support their autistic patients [28].

Additional patient-level factors: Compared to neurotypical individuals, autistic individuals have significantly lower healthcare self-efficacy [11]. For instance, autistic patients reported challenges with body awareness and articulation of internal sensation [37]. They also face difficulty judging if their symptoms warrant medical evaluation and participating in healthcare-related discussions [28]. Fear of perceived stigma could be a contributing factor: as autistic individuals expressed fear that their physicians might not take them or their complaints seriously [19]. Additionally, challenges with executive function and skills affect autistic individuals' healthcare experiences and outcomes in many ways. Autistic patients might find it difficult to prioritize their health issues, plan for future appointments, remember appointment details, remember to take their medications, and summarize their medical complaints and issues [19,28,37]. Fixated interests might affect healthcare encounters, particularly if focused on health information or related to a medical instrument or environment [56]. Possible behavioral challenges (i.e., shutdown or meltdown) might also hinder medical care and should be prevented, recognized early, and managed effectively [40]. Furthermore, associated emotional and psychiatric co-occurring conditions play a major role, with anxiety frequently reported as a barrier to healthcare that should be addressed [28,37]. Anxiety during healthcare encounters might be related to different factors including unpredictability, overstimulating sensory input, and previous traumatic medical experiences [33]. In addition to anxiety, gender diversity and greater disability are associated with more challenges with healthcare among autistic individuals [24]. Finally, the need for social support which might not always be available, accessible, or allowed could complicate healthcare access and delivery [19,37].

Provider-Level Factors

Provider's lack of knowledge and/or stereotyped beliefs about autism: Provider-related factors include lack of knowledge and low confidence and self-efficacy in providing care to autistic individuals [28,37]. In addition, physicians might have stereotyped views or negative attitudes toward autism leading to perceived stigmatization [28,37]. Healthcare providers across disciplines report limited knowledge and lack of adequate training about autism [25,36-38,42]. In the United States, the percentage of healthcare professionals working in adult medicine and obstetrics rating their ability to provide care to autistic patients as poor or fair exceeds 75% [48]. In a more recent study in the United Kingdom, approximately 40% of general practitioners reported having no proper training about autism with limited confidence in their abilities to care for autistic patients despite good fundamental knowledge [57]. In a study focused on caring for autistic children, nearly half of surveyed healthcare providers reported concerns related to their level of confidence in providing care to the autistic population [58]. This pattern is also seen among allied healthcare professionals such as child life specialists [59] and radiology technicians [60].

Limited knowledge and stereotyped assumptions about autism among healthcare providers can hinder optimal care in several ways [37]. For instance, providers might lack the specialized information required to screen for autism or refer for services, leading to delayed or missed diagnosis [31]. Providers might also question the autism diagnosis based on an apparent high level of functioning or label symptoms as psychosomatic without full evaluation [39]. Additionally, providers' behaviors and attitudes might add to the communication challenges facing autistic individuals in the healthcare system. For example, providers might rely on non-verbal means of communication when gathering information from autistic patients leading to misunderstanding and confusion [39]. Ultimately, miscommunication can hinder rapport and trust-building [48]. Correspondingly, in a survey exploring autistic patients' experience with healthcare, less than half of autistic respondents reported a good relationship with their doctor [19]. Consequently, autistic individuals might avoid the healthcare system for fear of stigma or misjudgment [31]. Similarly, physicians might lack motivation to spend time with patients who are considered to be challenging which could affect the quality of care provided [61]. Providers might underestimate autistic patients' cooperation and ability to tolerate medical procedures and overestimate failure rates [62], which might lead to giving up on essential investigations and interventions. Likewise, misconceptions about the sexuality of autistic patients might be a contributing factor to the lower rate of pap smear screening among this population [31].

Training to improve knowledge about autism and attitude toward autistic individuals is essential to attaining autism-friendly healthcare [43]. In a UK-based survey, access to clinicians with a good understanding of autism has been reported as the most important adjustment to healthcare to accommodate autistic individuals [35]. Similarly, a survey exploring the opinions of autistic individuals and their families showed that the education of healthcare providers about autism is a top priority [63]. Remarkably, 100% of surveyed healthcare providers working in a day hospital specialized in caring for children with neurodevelopmental disabilities, physicians, and allied health professionals expressed interest in additional training about autism [62]. Fortunately, training has been shown to be feasible and effective with previously reported successful programs targeting healthcare personnel across disciplines and professions including clinicians [64], administrative staff [64], security personnel [65], and ED workers [63]. However, as not all educational activities are perceived to be useful [57], previously reported successful strategies such as case scenarios and videos should be utilized [63,64]. Additionally, ASD training resources such as brochures and flyers should be available and accessible to enhance the confidence of professionals working with this population [66]. Moreover, a first-person perspective is recommended and appreciated by professionals interested in learning more about autism [59,63]. While autism-specific training might include variable topics, training should focus on previously identified gaps in knowledge, such as the heterogeneity of autism [39], accommodation strategies in different settings and how to implement them [67], available resources and how to access them [28], and management of patients affected by profound autism (non-speaking or have aggressive behaviors) [59]. Regardless of content and method, educational activities and training initiatives should be guided by a systematic needs assessment [66] and customized to meet the needs of the targeted audience [64]. Professional training could also be a part of a larger plan to transform a healthcare setting into an autism-friendly space alongside other interventions [55].

System-Level Factors

System-level factors impacting autism-friendly healthcare include logistical and operational factors, financial and economic factors, and social and societal factors [25,28,31,36,37]. Autistic patients have reported that the healthcare system is complex and hard for them to navigate without assistance [36,37]. Nonetheless, help, formal and informal, might not be available or accessible [36,37,40]. Caregivers of autistic adults have expressed a great need to simplify the processes required to access care in order for their children to achieve autonomy [33]. Additionally, the cost of healthcare utilization can prevent autistic individuals, similar to patients with other disabilities, from accessing care [26]. Fortunately, mandating insurance to cover certain services showed an increase in the use of these services by autistic patients [31]. However, caregivers of autistic individuals frequently report dissatisfaction with insurance [33]. Financial incentives can also cause providers to offer optimal care to autistic patients which might require extra time and effort [25]. General stigma about autism can stop autistic patients from accessing healthcare or worsen their overall healthcare experience [19,37]. For instance, autistic patients tend to worry that unfamiliar behaviors such as stimming might provoke reactions from other patients [19]. They also expressed concerns that adjustments aiming to facilitate their healthcare could be stigmatizing [28]. Finally, the healthcare system tends to depend on patients' social support (e.g., family members or friends) to assist with certain aspects of the healthcare experience (e.g., the discharge process) while limiting their involvement with other aspects (e.g., during outpatient visits), which might not exactly match the autistic patient's need for or availability of social supports [19,40]. Offering formal support to autistic patients to help with navigating the healthcare system and/or community efforts to help autistic individuals with social connection and building relationships could be helpful. 

Principles of change

Affirming findings from previous research, a recent exploratory study based on professional experts' and self- and family advocates' consensus included the following components of autism-friendly healthcare: flexibility to accommodate the unique needs of autistic individuals, direct involvement of autistic patients in their care, and notification of healthcare personnel involved in caring for autistic individuals about the diagnosis and its consequences prior to encounters [30]. Thus, for successful transformation into autism-friendly care, three key principles should be adopted to guide the transformation: proactivity and advanced planning, flexibility and individualization, and collaboration. 

Proactivity and Advanced Planning

Although adjustment should be offered based on individual patient's needs, an official autism diagnosis can help with advocating for adjustment [28]. Healthcare providers across disciplines have reported on the advantage of learning about the autism diagnosis as early as possible to facilitate medical encounters [59,60]. Thus, advanced planning should start with the early identification and correct diagnosis of autism [68]. However, autistic patients might avoid disclosure of diagnosis for fear of judgment and stigmatization [69-71], and physicians might underestimate the number of autistic patients under their care, even when the diagnosis is documented [48]. Thus, if adjustments seem to be required, person-centered accommodations should be initiated regardless of the formal diagnosis, particularly as self-identified autistic individuals report similar barriers to healthcare access and negative experiences and outcomes to those formally diagnosed individuals [19,68].

Once identified, patients might benefit from a flagging system that can communicate information regarding the autism diagnosis in an efficient nonstigmatizing manner [43,46]. Required adjustments should also be communicated as early as possible. Previously endorsed methods to achieve this goal included registration cards to be completed at the time of the visit [43], hidden disability lanyards [46], door signs [59], additional screening questions at the time of admission [72], and adding an alert to the electronic healthcare record [60].

Healthcare organizations and institutions should take measures to evaluate their readiness to provide care for autistic individuals and determine the most frequent and most severe barriers encountered by their clients [73]. This can be achieved by using previously developed tools and instruments such as the Barriers to Healthcare Checklist (short and long forms) [26] and Barriers to Providing Healthcare Measurement Tool [67]. This specific understanding allows for the prioritization of adjustment efforts to match local needs and priorities [73].

Flexibility and Individualization

While adjustments to prepare the healthcare system to accommodate the needs of the autistic population are recommended, these needs are heterogeneous [28,35,46]. For instance, sensory sensitivities and preferences vary significantly among autistic individuals suggesting that a person-centered approach to sensory optimization is critical [40]. Similarly, while some autistic adults appreciate advanced information gathering before medical encounters, others expressed frustration and experienced overwhelm when asked for a lot of information before the visit [46]. Thus, flexibility and individualization of care are required to achieve autism-friendly care. Both healthcare providers and systems should adopt a flexible approach to autism-friendly adjustment. On the contrary, the rigidity of the system or providers is frequently reported as a major challenge [59].

At the provider level, flexibility can be expressed through adjusting the communication style to match the different preferences among autistic individuals [28]. Additionally, providers can modify certain aspects of care to better match the needs of an autistic patient if the alternative is considered safe and appropriate (e.g., spot checks of vital signs instead of continued monitoring or use of non-traditional locations or positions for examination) [33,72]. An adaptable responsive provider was recognized as an important feature of a good healthcare experience by autistic adults in the United Kingdom [35]. At the system level, flexibility is required with procedures and processes such as offering multiple means of booking appointments, allowing longer duration for encounters, and amendment of rules regarding supporters allowed to accompany patients during an encounter [35,46].

Collaboration

Collaboration among different stakeholders at different levels is essential for the successful transformation to an autism-friendly healthcare system, which should entail collaboration with autistic patients and their families, collaboration between healthcare providers across specialties and disciplines, and larger-scale collaboration with other establishments providing services to the autistic population, such as the educational system and other community support systems [25].

Collaboration with autistic individuals and their caregivers should be prioritized at the clinical individual-care level, organizational quality-improvement level, and systemic level [24,25]. For instance, collaboration with parents when managing autistic children and youth has been endorsed by both families and providers as a requirement for positive healthcare experiences [25,59,72]. Providers can ensure collaboration with parents by acknowledging them as experts in their children and empowering them to participate in the evaluation process and care planning [25]. Beyond clinical care, policies and procedures guiding the management of autistic individuals should be co-designed with and evaluated by the autistic population [24]. Moreover, research to improve healthcare for autistic individuals should employ a community-participatory approach for better results [26].

At the provider level, the complexity of providing care to autistic individuals beyond usual roles has been previously reported [25]. Additionally, a lack of coordination among providers was endorsed as a significant barrier to optimal care for autistic patients [67]. Thus, collaboration through open clear communication, coordination of care, and cooperation among healthcare providers with different expertise is required for autism-friendly healthcare [41]. This could be achieved in various ways, such as working within an interprofessional team [25], care coordination [25], or appointment of champions to facilitate collaboration [45]. Well-designed collaboration efforts can particularly target specific gaps in autism care as evidenced by the Extension for Community Healthcare Outcomes (ECHO) Autism, which supports the early identification, diagnosis, and management of autistic individuals through collaboration between experts and community providers [31]. Similarly, ECHO Autism has been shown to support young autistic adults at the time of transition from pediatric to adult care [74]. Partnerships to connect with educators and other related services can be beneficial, particularly when designing accommodation strategies or adapting the environment to autistic patients' needs [45].

Intervention strategies

Guided by the principles of care adjustment discussed above, various strategies could be designed and applied, individually or combined, to improve the healthcare experience for autistic patients. We discuss here examples of previously reported intervention strategies with promising results. However, further research is required for improvement and generalization.

Data Collection and Communication Tools (Health Passport/Hospital Passport)

Hospital passports are tools designed to document and communicate important health-related information to facilitate care, with demonstrated efficacy among individuals with intellectual and developmental disabilities [75]. While additional empirical work might be required to support its use for autistic patients, preliminary results are promising [45]. For instance, the Quick Tips Card (QTC), designed by Bultas et al. to facilitate the care of autistic children in office-based settings, showed high rates of accessibility and usefulness among caregivers and healthcare providers [58]. The card contained relevant healthcare and autism-related information including preferred communication method, special interests, calming techniques, sensory needs, and triggers to equip providers with the necessary information to ensure successful encounters. Caregivers can document additional comments and requests which could empower them to advocate for their children and their needs [58]. Other similar tools have been designed to facilitate inpatient hospitalization of autistic patients, which could be filled before or during admission [76,77]. In addition to gathering information regarding receptive, expressive, and pragmatic communication skills and challenges, the autism-specific care plan (APC) collects information related to safety which is particularly relevant for inpatient encounters [76].

For autistic adults, the Autism Healthcare Accommodations Tool (AHAT) was designed by the Academic Autistic Spectrum Partnership in Research and Education (AASPIRE) using a community-based participatory research approach to document patients' preferences and generate a personalized accommodation report [78]. The patient or his caregiver can complete the AHAT survey online to automatically create a report with actionable, patient-centered recommendations for adjustment. AHAT report includes recommendations related to scheduling appointments, waiting room accommodations, preferred communication styles, sensory issues, and accommodations to facilitate follow-up care. It also documents the need for supporters and their expected roles in care [78]. The AHAT is intended to be used as a part of AASPIRE Healthcare Toolkit which includes additional worksheets and resources for patients and providers; the full toolkit is available at http://autismandhealth.org [78]. Preliminary evaluation data showed highly rated practicality and satisfaction with reported decreased barriers to care and improved healthcare self-efficacy among autistic patients after using the toolkit [78]. A more recent preliminary study exploring the experience of Australian autistic patients using the AASPIRE Healthcare Toolkit supported its use to facilitate communication between patients and providers particularly for newly diagnosed patients and less experienced providers with emphasis on the need to modify the toolkit to include Australia-specific information and resources [79].

Package of Interventions

Package of intervention refers to variable approaches that encompass multiple strategies grouped and applied together with the goal of identifying patients with certain healthcare needs and accommodating them to ensure universal adherence and minimize provider-to-provider variations such as clinical pathways or algorithm-based interventions [45]. For instance, to optimize care for hospitalized autistic adults, a collective effort of multiple autism experts, healthcare providers, autistic patients, and their families at the Massachusetts General Hospital (the MGH Autism Care Collaborative) developed a package of interventions including a data collection tool (Autism Care Questionnaire), a clinical pathway (Clinical Care Algorithm), and a standard checklist for orders and accommodation (Admission Basic Checklist) [80]. The main goal of the Autism Care Questionnaire was to survey autistic patients and their caregivers to identify their preferences regarding communication and care adjustment. On the other hand, the Admission Basic Checklist’s primary goal was for providers to have a protocol for the types of orders, consultants, and adjustments that should be considered when admitting a patient with ASD [80].

Similarly, Children's of Alabama in Birmingham introduced a clinical pathway (known as "Sensory Pathway") to be used during acute hospital visits (emergency or inpatient encounters) when a patient with sensory differences is identified [55]. The pathway was a result of a collaborative effort with multiple stakeholders including patients and their families and included the following components: training, provision of sensory toolkits and storyboards, early involvement of allied healthcare professionals, and early and continuous collaboration with parents and caregivers. The pathway could be activated anytime during the encounter by healthcare professionals, patients, or families. A brief screener was employed to identify patients with sensory needs but was not essential for activating the pathway. When piloted, 48% of the patients who used the pathway had an ASD diagnosis [55]. Evaluation data showed positive results based on families' responses. Of note, the sensory toolkit was rated as the most beneficial step of the pathway [55]. 

Specialized Health Check or Primary Care Visits

A multi-component primary care or health check visit designed specifically to support adult autistic patients has been proposed to optimize their care [46]. Similar to health check programs which have been utilized to support adults with intellectual disabilities [81], the goal is to adopt a proactive approach, identify potential gaps in care, and accommodate special needs. The planned health check visit incorporates a pre-appointment questionnaire (PAQ) (to be completed by patients before the encounter) and a health check template (to be used by clinicians during the encounter) with preliminary support from autistic individuals and their caregivers [46].

Medical Home or Specialized Primary Care Facilities

The medical home model has proven to be necessary and effective to provide comprehensive, evidence-based, high-quality care for all children, with and without special healthcare needs [82,83]. Nonetheless, autistic children are less likely to have an established medical home [84-86]. When studied, autistic children who received care through medical home intervention were more likely to have fewer unmet needs, be more satisfied with care, and participate in shared decision-making compared to autistic children who continued to receive care through a standard primary model [87]. The medical home model is particularly equipped to meet the needs of autistic children if it prioritizes collaboration with parents and invests in autism-specific education for healthcare providers [88]. Special focus of medical homes should be used to improve access to care for autistic adolescents approaching the transition into adult care [89].

For autistic adults, a specialized primary care facility might provide easier access to professionals who are better equipped to manage autistic patients with an enhanced sense of community [33]. The Center for Autism Services and Transition (CAST) adopted a patient-centered medical home (PCMH) model in collaboration with autistic advocates to enhance the primary care of autistic adults, especially preventative care through ongoing prevention, routine medical management, and care coordination [90,91]. Evaluation studies showed that CAST autistic patients have better continuity of care, higher satisfaction, and improved preventive care [90-92]. CAST has been modifying and improving its services to match the needs of the autistic population [93]. For example, "happy visits" are offered for desensitization and increasing tolerance of the medical office. Similarly, procedure videos have been produced to demonstrate common procedures such as blood pressure measurement and vaccination [93].

Environmental and Architectural Design

Environmental design according to the particular needs of the autistic population can lead to autism-friendly spaces with enhanced accessibility and experience. In the healthcare setting, this can be achieved through meticulous forward-thinking planning and iterative assessment and adjustment. First, an accessible location in a quiet neighborhood should be selected [35,40,53]. An optimized sensory environment with minimized stimuli and details and enhanced transitional experiences is required to address autistic individuals' variable hypo- and hypersensitivities [51,53]. Additionally, the built environment should have a clear layout with a simple spatial organization that promotes predictability and addresses the autistic patient's need for sameness and difficulty handling change [53]. For example, visual tools such as pictures, pictograms, and color coding should be employed to specify the purposes of different spaces and facilitate wayfinding [51,53]. Furthermore, the built environment should prioritize safety with appropriate security measures, particularly for autistic children or adults who might not apprehend danger. Different frameworks and approaches have been proposed to consolidate these strategies and facilitate their application in different settings [10,94].

Autistic SPACE is a novel framework designed to represent the barriers and solutions of the autistic experience in healthcare settings using a memorable acronym (S for sensory needs, P for predictability, A for acceptance, C for communication, and E for empathy) [10]. This framework aims to fulfill autistic patients' needs for a "wider" physical, processing, and emotional space to achieve an ideal healthcare experience. The wider physical space will address the anxiety and discomfort that might result from proximity to others. A longer-than-typical processing space is required for autistic patients to manage new information and unanticipated changes in order to participate successfully in their healthcare-related discussions and decision-making. The emotional space, which should include physical spaces and time for recovery, will facilitate the recognition and management of emotions before escalation to behavioral challenges [10].

The healthcare environment could also be evaluated and adjusted to accommodate autistic patients' needs based on MacLennan et al.'s work which advocates for six principles to determine the suitability of environments to the autistic population's needs [94]: the "sensoryscape" which encompasses the nature and intensity of sensory input; space constraints which include building confinement and/or overcrowding; predictability, which means available, accessible, and consistent information and plans; understanding from others, which includes replacement of judgments with support; adjustments, which include modification for the pace and communication style; and opportunity for recovery, which include spaces to prepare for and/or recover from certain aspects of care [94]. A structured checklist can be used to guide the evaluation and adjustment of healthcare environments to meet the needs of autistic patients.

Behavioral Strategies

Behavioral strategies can effectively facilitate medical encounters for autistic patients [45]. Reinforcement of expected behaviors, pairing, giving choices to allow for more control, using "first/then" statements, creating a behavioral momentum, peer modeling, visual tools (e.g., visual timers/schedules), verbal tools (e.g., verbal countdown), and distractions can be used to facilitate healthcare visits and procedures [56,95]. Autism Speaks has published several toolkits to help providers utilize behavioral strategies to facilitate medical procedures such as electroencephalogram and blood draws, which can be accessed online (https://www.autismspeaks.org/autism-speaks-tool-kits). 

Technology-Based Strategies

Technology can be utilized to apply and enhance previously discussed behavioral strategies. For example, social stories and scripts can be more accessible if available in an electronic format. In an exploratory study, "Going to Imaging," a social script intervention application was shown to lower anxiety, decrease challenging behaviors, and reduce the time needed to complete imaging procedures among autistic children [96]. Parents of autistic children also reported using their phones to take photos of their doctors and show them to their children to prepare for medical visits [56]. Additionally, to overcome tactile aversion when measuring vital signs, a Fitbit could be used instead of traditional tools [33]. Moreover, technology, such as tablets, can be used strategically to cope with waiting time or unfavorable medical procedures and settings [33]. Immersive virtual reality (VR) is increasingly used as a distraction tool to facilitate medical procedures [97,98] with previously reported success. Recently, an exploratory study introduced social robots (small humanoid NAO and the pet-like MiRo) to facilitate simulated medical encounters for autistic children with encouraging initial results [99]. Furthermore, telehealth might be considered, as it can eliminate stress related to unwanted socialization and sensory overstimulation in the clinical environment [100].

## Conclusions

Autism-friendly healthcare is a person-centered model of care focused on facilitating and improving healthcare access and delivery among this vulnerable population. Adaptation of an autism-friendly transformation of healthcare is important to improve access and delivery. The components of autism-friendly healthcare based on this review are summarized in Table 1. The first step toward autism-friendly healthcare is to address current obstacles and barriers as reported by autistic patients, their caregivers, and healthcare providers. These barriers include patient-level factors, provider-related factors, and system-level factors. Advanced planning, collaboration among important stakeholders, and flexibility are essential to guide the transformation of the healthcare system. Multiple interventional strategies (e.g., hospital passport, sensory coping kits, specialized care visits, behavioral strategies, and technology) have been implemented in different healthcare settings with promising results. Additional work is required for optimization and generalization.
